# Three-Dimensional Hierarchical Porous TiO_2_ for Enhanced Adsorption and Photocatalytic Degradation of Remazol Dye

**DOI:** 10.3390/nano11071715

**Published:** 2021-06-29

**Authors:** Jitpisut Poolwong, Tanya Kiatboonyarit, Supakit Achiwawanich, Teera Butburee, Pongtanawat Khemthong, Sutasinee Kityakarn

**Affiliations:** 1 Department of Chemistry, Faculty of Science, Kasetsart University, Bangkok 10900, Thailand; jitpisut.liw@gmail.com (J.P.); ky.501@hotmail.com (T.K.); supakit.a@ku.th (S.A.); 2 National Nanotechnology Center (NANOTEC), National Science and Technology Development Agency (NSTDA), 111 Thailand Science Park, Klong Laung, Pathumthani 12120, Thailand; pongtanawat@nanotec.or.th

**Keywords:** textile dye degradation, photocatalysts, hierarchical porous TiO_2_, 3DOM, inverse opal structure

## Abstract

Three-dimensional hierarchical mesoporous structures of titanium dioxide (3D-HPT) were synthesized by self-assembly emulsion polymerization. Polymethyl methacrylate (PMMA) and pluronic 123 (P123) were used as the soft templates and co-templates for assisting the formation of hierarchical 3D porous structures. The TiO_2_ crystal structure, morphology, and Remazol red dye degradation were investigated. The 3D-HPT and normal three-dimensional titanium dioxide (3D-T) presented the good connection of the nanoparticle-linked honeycomb within the form of anatase. The 3D-HPT structure showed greatly enhanced adsorption of Remazol dye, and facilitated the efficient photocatalytic breakdown of the dye. Surprisingly, 3D-HPT can adsorb approximately 40% of 24 ppm Remazol dye in the dark, which is superior to 3D-T and the commercial anatase at the same condition (approx. 5%). Moreover, 3D-HPT can completely decolorize Remazol dye within just 20 min, which is more than three folds faster than the commercial anatase, making it one of the most active photocatalysts that have been reported for degradation of Remazol dye. The superior photocatalytic performance is attributed to the higher specific surface area, amplified light-harvesting efficiency, and enhanced adsorption capacity into the hierarchical 3D inverse opal structure compared to the commercial anatase TiO_2_.

## 1. Introduction

The dyeing process is one of the major steps in textile industries. This process releases a lot of wastes containing both organic dyes and chemicals into the environment [1,2]. Normally, the synthetic organic dyes in the textile industries are the azo compounds that are mutagenic, toxic, and carcinogenic [3]. These harmful organic molecules cause severe pollution to the aquatic ecosystem. Therefore, efficient and cost-effective removal of these toxic wastes is very important. In the recent years, dye removal and wastewater treatments by physical [4,5], chemical [6,7] or biological [8] methods have been developed [9]. In particular, photocatalytic technology based on semiconductor materials provides an effective way to control environmental pollution [2,10]. Among semiconductor photocatalysts, titanium dioxide (TiO_2_) has been widely used in photocatalytic degradation of pollutants because of its respectable photocatalytic properties, chemical and biological inertness, no secondary pollution, and low cost [6,11]. There have been numerous reports about using TiO_2_ and its composites in various photocatalytic environmental remediations such as waste degradation [1,11,12,13], water and air purification [7,11,14], carbon dioxide reduction [15,16,17,18], conversion of biomass-derived wastes to valuable chemicals [19,20,21], and photocatalytic water splitting for alternative hydrogen energy [22,23,24,25,26,27,28,29]. However, the main drawbacks of unmodified TiO_2_ are the undesirable recombination of electrons and holes, the low efficiency under visible light irradiation, and the limited adsorption of dyes on the catalyst’s surface. The photocatalytic performance of TiO_2_ could be improved by chemical modification or nanostructural designs [11,24,26,30,31,32,33]. 

Rational designs of nanostructures generally involve nanoscale building blocks on the surface of materials [30,34,35]. For example, 0D-3D TiO_2_ nanostructures showed significantly enhanced photocatalytic performance [24,25,31,33,34,36,37,38,39]. In particular, 3D structures can enlarge the specific surface area, resulting in high photodegradation capability [11,24,32,33,40,41,42,43,44]. For instance, Zhang et al. prepared Au-doped three-dimensional macro-porous (3DOM) TiO_2_ that show high photocatalytic performance, as indicated by complete decolorization of Methylene Blue (30 mg/L) within ~60 min [45]. Ma et al. reported the Bi_2_S_3_/3DOM TiO_2_ composite exhibiting impressive photocatalytic degradation of Rhodamine B within (10 mg/L) within approx. 360 min [46]. 3D-TiO_2_ can be synthesized by sol-gel, solvothermal, self-assembly, and template-assisting methods [26]. The resulting inverse opal structures could effectively enhance absorption and multiple reflections of light in the material [26,42,43,44]. Moreover, a double templating technique has attracted considerable interest as it provides additional mesopores, forming hierarchical structures with rich functionality such as increased surface area, improved solar absorption [22,33] improved charge transfer ability [11], and enhanced dye adsorption [11,22,26,34,47]. Introduction of mesopores inside the conventional macroporous 3DOM could lead to hierarchical nanostructures with enhanced dye adsorption and overall photocatalytic performance. Conventionally, silica [35], poly(methyl methacrylate) (PMMA) [31,34,40,47,48], and polystyrene [26,41] spheres are the popular templates used for fabricating the 3DOM structures, while pluronic 123 is known as the effective soft template to create regular mesopores [24]. 

In this work, we adapted the techniques to synthesize macro-porous 3DOM and mesoporous together, creating the novel hierarchical 3DOM TiO_2_ (3D-HPT) photocatalyst with unusually high photocatalytic performance for textile dye degradation. The catalyst was prepared using PMMA and pluronic P123 as the macropore and mesopore templates, respectively. The resultant nanostructure significantly increases dye adsorption efficiency compared to the normal TiO_2_ 3DOM (3D-T), as it can enable adsorptive removal of approximately 40% of Remazol Brilliant Red 3BS dye in dark, while 3D-T does not show this property. Moreover, the catalyst can decolorize the dye within 20 min under simulated sunlight irradiation (AM 1.5G), which is more than two times quicker than the normal 3DOM (approximately 40 min), making it one of the most active non-metal-doped photocatalysts that have been reported for degradation of Remazol dye.

## 2. Materials and Methods

### 2.1. Preparation Methods and Characterizations

PMMA macrospheres were prepared by following the method reported in our previous works [31,48]. PMMA templates were allowed to self-assemble by emulsion polymerization of methylmethacrylate monomer (MMA). 2,2′-azobis (2-methylpropinoamide) (≥97%, Wako, Japan) was used as an initiator, and the PMMA colloidal was synthesized under N_2_ at 65 °C for 3 h. The sedimentation of the crystal colloidal PMMA was performed by naturally evaporation of the solvent for 2 months at room temperature. 

In a typical synthesis of 3D-HPT, 3 g of titanium isoproproxide (≥97%, Sigma-Aldrich, Bangalore, India) was mixed with 0.6 g of pluronic P123 (Sigma-Aldrich, St. Louis, MO, USA, MW = 5800), 1.4 g of 38% HCl (Carlo Erba, UK), 0.46 g of 44 wt% H_2_SO_4_ (>95%, Merk, Taufkirchen, Germany), and 30 g of absolute ethanol (99.5%, MACRON, USA). Pluronic P123, a tribock copolymer which is reported as an effective agent to create tiny pores on various metal oxides and metal-organic materials, was intentionally added into the system in order to induce additional micro/meso pores in the conventional 3DOM structures. The mixture was stirred for 20 h at 40 °C. The solution was then dropped into the dried crystal colloidal PMMA, and evaporated at 40 °C under an ambient condition for 2 days. Then, the samples were heated up to 100 °C for 2 days to allow the formation and stabilization of the 3D network. Afterward, the as-prepared powders were calcined at 450 °C in a muffle furnace with a ramp rate of 3.0 °C/min under air atmosphere for 4 h. In this step, the macro-porous (PMMA) and meso/micro-porous (P123) templates were simultaneously removed. After cooling down naturally to room temperature, 3D-HPT that consists of both macro and meso/micro pores in the structure was obtained. The conventional 3DOM TiO_2_ (3D-T) was prepared in a similar approach, except there was no pluronic 123 added to the precursor solution.

Crystal structures of the samples were analyzed by X-ray powder diffraction (XRD) using a Bruker D8 Advanced X-ray Diffractometer (Germany) with Cu K_α_ radiation at a scanning range from 20 to 70° with a step size of 0.02°. The characteristic peaks were confirmed by the Powder Diffraction File (PDF) database. The morphology of the structures was observed by field-emission scanning electron microscopy (FESEM, Hitachi SU8030-FESEM, Japan, operated at 10–15 kV) and transmission electron microscopy (TEM, JEOL2100 Plus, Japan, operated at 200 kV). The specific surface area was determined by a Quantachrome, NOVA e-Series Surface analyzer (USA) using the Brunauer–Emmett–Teller (BET) method with nitrogen adsorption apparatus. The ultraviolet (UV)–visible reflectance was determined by a UV–visible spectrophotometer (PerkinElmer, Lamda365, UK), and the degradation of Remazol Brilliant Red was determined by a UV–visible spectrophotometer (Shimadzu, UV-1601, Japan).

### 2.2. Photocatalytic Studies

The photocatalytic reactivity of the samples was investigated by degradation of Remazol Brilliant Red under simulated solar irradiation. The reaction was carried out in a beaker wrapped by an outer jacket made of aluminum foils. In the general procedure, 17 mg of the catalyst was suspended in 90 mL of aqueous Remazol Brilliant Red solution (24 ppm) under mechanical stirring in dark for 30 min. Then, the mixture was irradiated by a solar simulator equipped with the AM 1.5G filter (Newport LCS-100, 100W, USA). We collected 5 mL of the solution from the reaction bath at a regular interval. The sampled solutions were filtrated to remove the suspended catalyst particles and then analyzed by an UV–visible spectrophotometer (Shimadzu, UV-1601). The degradation of Remazol Brilliant Red was monitored by measuring the absorbance at λ_max_ 540 nm. For comparison, commercial anatase powder (≥99%, Sigma-Aldrich, St. Louis, MO, USA) was also tested under an identical condition.

## 3. Results and Discussion

### 3.1. Characterization of TiO_2_ Photocatalysts

**Figure 1:**
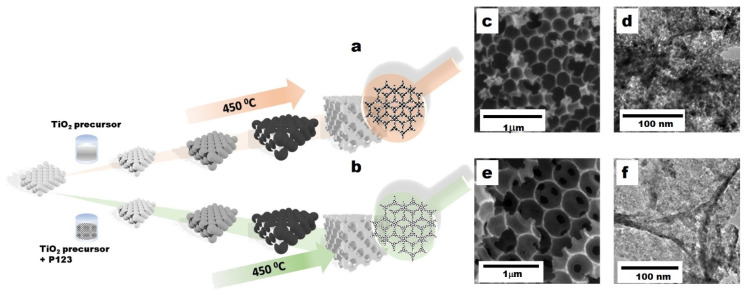
(**a**,**b**) Schematic illustration showing the synthetic procedures of three-dimensional titanium dioxide (3D-T) and three-dimensional hierarchical mesoporous structures of titanium dioxide (3D-HPT). (**c**,**e**) Scanning electron microscopy (SEM) images of 3D-T and 3D-HPT, respectively. (**d**,**f**) the corresponding transmission electron microscopy (TEM) images of 3D-T and 3D-HPT, respectively.

The synthetic procedures of 3D-T and 3D-HPT are shown in Figure 1a,b, respectively. The TiO_2_ precursor solution was allowed to impregnate into the void of the hexagonal close-packing of crystal colloidal PMMA. During the increasing temperature, the colloidal self-assembly of PMMA spheres turned to the close-packing carbon bead, which acts as a template of the 3DOM structure. The temperature reached the glass transition state producing the TiO_2_-glass state-3DOM framework. After calcination at 450 °C under an oxidizing atmosphere, the interconnected TiO_2_ particles formed the 3DOM structure, simultaneously with the combustive removal of the PMMA template. The highly uniform mesopores in the 3D-HPT duplicated from the monodisperse emulsion droplets of pluronic P123 (P123) under acidic conditions. The morphology of the samples was observed by SEM and TEM. The SEM images of 3D-T and 3D-HPT show that both have a similar microstructure which is constructed with well-ordered macropores (Figure 1c,e). However, careful investigation of the structure at higher magnification by TEM (Figure 1d,f) revealed that 3D-T is constructed by the larger TiO_2_ particles, compared to that of 3D-HPT, indicating that the pluronic 123 template could induce finer grains and pores in the TiO_2_ interconnection wall. This has a great influence on the adsorption of dye and the overall photocatalytic performance. 

**Figure 2:**
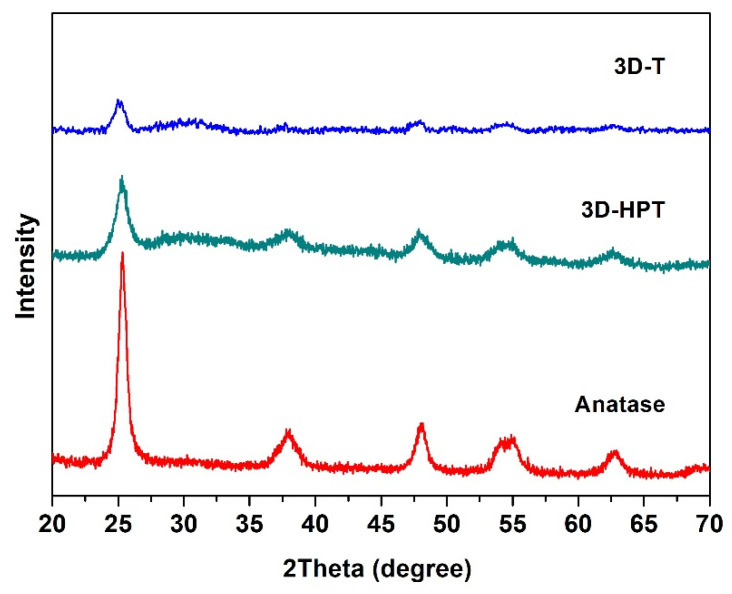
X-ray diffraction (XRD) patterns of the commercial TiO_2,_ 3D-T, and 3D-HPT catalysts.

**Figure 3:**
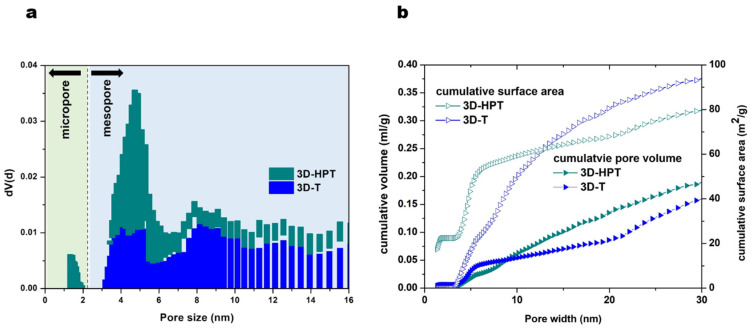
(**a**) Pore size distribution and (**b**) cumulative of pore volume and surface area of 3D-T and 3D-HPT catalysts.

The crystallinity of the commercial TiO_2_ (anatase), 3D-T and 3D-HPT was characterized by using X-ray diffraction (XRD) (Figure 2). The XRD patterns of all samples show the diffraction peaks at 25.3°, 37.8°, 47.6°, 54.1°, and 62.6°, corresponding to the anatase crystalline phase, which is in well agreement with the Powder Diffraction File (PDF Number 21-1272). XRD patterns of 3D-T and 3D-HPT show broader peaks, indicating the smaller particle size of TiO_2_ in the 3DOM structures compared to the commercial TiO_2_. The specific surface area and pore size of the commercial anatase TiO_2_, 3D-T, and 3D-HPT were analyzed by N_2_ adsorption, as shown in Table 1. The specific surface area of 3D-T and 3D-HPT was in the range of 105.5–109.6 m^2^/g, which is significantly higher than that of the commercial anatase TiO_2_ (96.0 m^2^/g). The average pore diameters of the commercial anatase, 3D-T, and 3D-HPT were 6.33, 4.04, and 3.40 nm, respectively. The higher specific surface area of the 3DOM structures is possibly due to the more regular nanoparticle arrangement of hexagonal pores in the structures, while anatase TiO_2_ does not have this feature. Interestingly, the pore size distribution of 3D-HPT shows two distinctive pore types in the ranges of 1–2 nm, and 3–7 nm (Figure 3a), which is the characteristic of the hierarchical structure. On the other hand, the micro-pore characteristic does not exist in the conventional 3DOM (3D-T) structure. The cumulative pore volume and surface area of 3D-HPT and 3D-T are shown in Figure 3b. The pore volume and surface area of 3D-HPT with the pore width less than 3 nm is obviously higher than that of the 3D-T due to the hierarchical mesoporous feature of 3D-HPT (Figure 3b). This property could result in the significant enhancement of dye absorption capacity of the 3D-HPT.

### 3.2. Photocatalytic Activities

**Figure 4:**
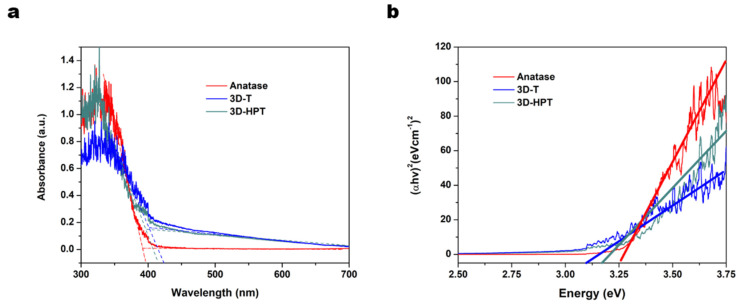
(**a**) Ultraviolet (UV)-visible absorption spectra, and (**b**) Tauc plot with the band gap energy.

**Figure 5:**
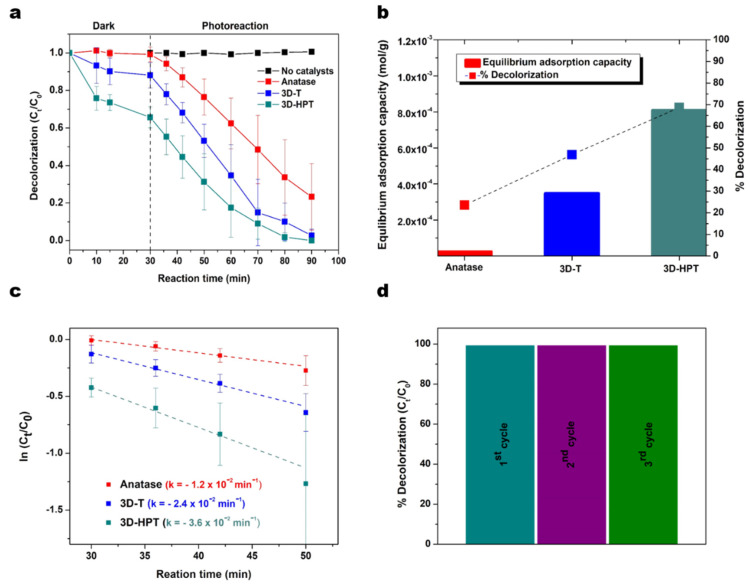
(**a**) Adsorption and decolorization of Remazol red dye under the dark, and light irradiation. (**b**) The equilibrium adsorption capacity and the percentage of decolorization at 20 min of photoreaction. (**c**) Rate of decolorization reaction derived from anatase TiO_2_ (red), 3D-T (blue), and 3D-HPT (dark cyan) catalysts. (**d**) Recyclability tests of 3D-HPT (showing percentage of decolorization of the dye after 60 min of phothocatalytic reaction). The reactivity remained unchanged after the repeated uses for three cycles.

The UV–visible absorption spectra of 3D-T, 3D-HPT show the red shift of absorption edge compared to the commercial TiO_2_, due to the 3DOM structure (Figure 4a) [39]. The band gap energy was calculated based on the Tauc plot with the equation:

(αhν)^n^ = A (hν − E_g_) (1)

where α is the absorption coefficient, hν is photon energy and relation constant, A is absorbance and E_g_ is optical band gap. In this study, the energy band gap of photocatalyst is calculated with the direct band gap n = 2. The energy gaps of 3D-T, 3D-HPT, and anatase TiO_2_ which were extrapolated from the Tauc plot were 3.10, 3.17, and 3.25 eV, respectively (Figure 4b). The adsorption and photocatalytic activities of 3D-T, 3D-HPT, and anatase TiO_2_ were evaluated against the photodegradation of Remazol red dye (24 ppm). The maximum absorption peak of Remazol red dye at 541 nm was used for calculating the adsorption and reaction rates (Figure 5a). The equilibrium adsorption capacity was calculated from the concentration of dye absorption on the catalysts at 30 min in dark conditions, as shown in Figure 5b. 

In the dark condition, the equilibrium adsorption capacities of 3D-HPT, 3D-T, and the commercial anatase were 0.81, 0.35, and 0.02 mmol/g, respectively. Interestingly, the absorption capacity of 3D-HPT was three times greater than that of 3D-T due to the smaller pores (Figure 3). Remazol red dye was obviously more adsorbed on the 3D-HPT than the other catalysts. This impressive result is possibly due to the mesostructure in 3DOM. Photocatalysts with high dye-loading capacity are also desirable for other applications such as dye-sensitized solar cells (DSSC), [49,50,51,52] highlighting the potential utilization of our developed catalysts in other applications. The photocatalytic efficiency of the 3D-HPT was also compared with the commercial anatase TiO_2_ and no catalysts, and the results are presented in Figure 5a. It was found that the catalysts with 3DOM structure (3D-T) exhibited a high photodegradation efficiency as it can decolorize Remazol red dye by approximately 82% within 60 min, while the commercial anatase TiO_2_ can decolorize just 65% at the same reaction time. This result shows that the increased specific surface area can greatly promote the dye removal performance [11,38,53]. 3DOM structure also enables multiple light reflection and light scattering, resulting in the increase of photoionization in the periodic macroporous structure [53]. Moreover, 3D-HPT showed the highest photocatalytic efficiency, as the complete decolorization of dye happened within just 50 min and the rate of the photoreaction was faster than 3D-T by about 1.5 times (Figure 5c). Moreover, 3D-HPT shows high recyclability, as the decolorization performance remain nearly 100% for three cycles of use, as shown in Figure 5d. Degradation was not observed with an absence of the catalysts (control experiment). Figure 5b (line) shows the photocatalytic performance of all catalysts at 20 min of photoreaction time, indicating that the highest photocatalytic performance was 3D-HPT. The results show that the mesoporous in 3D-HPT significantly promoted both the absorption of dye on the surface of the catalysts and the photocatalytic activity for degradation of Remazol red dye. 

The enhanced photocatalytic performance of TiO_2_ was attributed to the hierarchical porous structure of 3D-HPT, prepared by using PMMA as a macropore template and pluronic 123 as a mesopore template. First, the macroporous structures of 3D-HPT and 3D-T increase the specific surface area of materials compared to the commercial TiO_2_ nanoparticles. The inverse opal structure (IOS) of 3D-HPT and 3D-T synthesized using PMMA template (Figure 1c,e) shows the red shift of the absorption edge, indicating a slight reduction of the band gap energy (Figure 4a,b) of the 3DOM structures compared to the commercial TiO_2_. Moreover, the inverse opal structure of 3D-HPT and 3D-T also endows multiple light scattering through the spherical voids [39,40,41,42,43,44,54,55]. The increase of path length of light leads to light trapping in the IOS, which results in enhanced light harvesting efficiency. This phenomenon promotes the more efficient electron-hole pair generation [42,55]. Second, the mesopores with the finest grains in 3D-HPT not only promote the adsorption of the reactant, but also shorten the charge migration length. In other words, photogenerated charges need not migrate far to encounter the reactant. Therefore, charge transfer efficiency is significantly enhanced, and charge recombination is suppressed. These phenomena result in the enhanced photocatalytic efficiency of 3D-HPT. Notably, just simply adding the low-cost block co-polymer (pluronic P123) to the conventional synthesis process of 3DOM TiO_2_ can lead to dramatic enhancement of dye removal efficiency, highlighting that our developed strategy could be more economical compared to other methods such as noble-metal or transition-metal doping [45,56,57,58,59]. On the other hand, template-free synthesis should be of the focus of the future research, as 3D-HPT may still be unable to compete with the nanocrystalline anatase in terms of cost-effectiveness, although its catalytic performance is higher by many orders of magnitude. 

## 4. Conclusions

We demonstrated the facile synthetic strategy to create the hierarchical 3D-porous TiO_2_ nanostructure (3D-HPT) that showed high activity for removal of an aquatic dye. We prepared 3D-HPT by using PMMA and P123 as the macropore and micro/mesopore templates, respectively. TEM and SEM characterization revealed the successful formation of the inverse opal 3DOM structure, which had significantly higher surface area than that of the commercial TiO_2_. In addition, insightful investigation of pore-size distribution showed that the 3D-HPT consisted of a high density of micro/mesopores with an average diameter of 3.4 nm, while the conventional 3DOM structure synthesized by the similar condition (except no P123 added) did not have this character. Consequently, 3D-HPT exhibited dye adsorption capacity of 0.81 mmol/g, which was significantly higher than that of the conventional 3DOM structure and the commercial anatase (0.35, and 0.02 mmol/g, respectively). Moreover, the tiny pores inside the 3D-HPT also shortened charge migration length, suppressing charge recombination. Due to the synergistic effects of these properties, 3D-HPT exhibited superior photocatalytic performance for removal of Remazol red dye with a rate constant of 3.6 × 10^−2^ min^−1^, compared to the conventional 3DOM and the commercial TiO_2_ (anatase) which have the rate constant of 2.4 × 10^−2^ and 1.2 × 10^−2^ min^−1^, respectively. As high dye adsorption capacity and photocatalytic efficiency are desirable properties for other applications such as dye-sensitized solar cells, we hope that 3D-HPT could attract broad interest in photocatalytic research in the future. 

## Figures and Tables

**Table 1:** The specific surface area and the average pore size of the catalysts.

Catalysts	Surface Area (m^2^/g)	Pore Diameter (nm)
Commercial anatase	96.0	6.33
3D-T	109.6	4.04
3D-HPT	105.5	3.40

## Data Availability

Not applicable.
